# Whale Sharks, *Rhincodon typus*, Aggregate around Offshore Platforms in Qatari Waters of the Arabian Gulf to Feed on Fish Spawn

**DOI:** 10.1371/journal.pone.0058255

**Published:** 2013-03-13

**Authors:** David P. Robinson, Mohammed Y. Jaidah, Rima W. Jabado, Katie Lee-Brooks, Nehad M. Nour El-Din, Ameena A. Al. Malki, Khaled Elmeer, Paul A. McCormick, Aaron C. Henderson, Simon J. Pierce, Rupert F. G. Ormond

**Affiliations:** 1 Herriot-Watt University, Edinburgh, United Kingdom; 2 Qatar Ministry of Environment, Doha, Qatar; 3 UAE University, Abu Dhabi, United Arab Emirates; 4 Environment Department, University of York, Heslington, York, United Kingdom; 5 The School for Field Studies, Center for Marine Resource Studies, Turks & Caicos Islands; 6 Marine Megafauna Foundation/ECOCEAN USA, Tofo Beach, Mozambique; 7 Marine Conservation International, Edinburgh, United Kingdom; University of Sydney, Australia

## Abstract

Whale sharks, *Rhincodon typus,* are known to aggregate to feed in a small number of locations in tropical and subtropical waters. Here we document a newly discovered major aggregation site for whale sharks within the Al Shaheen oil field, 90 km off the coast of Qatar in the Arabian Gulf. Whale sharks were observed between April and September, with peak numbers observed between May and August. Density estimates of up to 100 sharks within an area of 1 km^2^ were recorded. Sharks ranged between four and eight metres’ estimated total length (mean 6.92±1.53 m). Most animals observed were actively feeding on surface zooplankton, consisting primarily of mackerel tuna, *Euthynnus affinis,* eggs.

## Introduction

The whale shark, *Rhincodon typus*, has a circumglobal distribution in tropical and subtropical oceans [1]. It is the world’s largest extant fish, yet there are still significant gaps in our understanding of its behaviour and ecology [2]. Furthermore, while of great popular interest and value to marine wildlife tourism [3], [4], populations have been impacted by fisheries throughout the Indo-Pacific [5]–[10] Whale sharks have therefore been classified as Vulnerable on the IUCN Red List of Threatened Species [11] and listed on Appendix II of the Convention on International Trade of Endangered Species (CITES) in 2002.

Whale sharks are known to feed on a variety of planktonic and nektonic organisms [1] by flexibly employing surface ram feeding, sub-surface filter feeding or stationary suction feeding [12], [13]. Whale sharks are known to aggregate seasonally in a number of areas, including Western Australia [14], Belize [15], Northern Mexico [16], Philippines [17], Djibouti [18], Mozambique [19], the Maldives [20], [21] and Seychelles [22], [23], usually in response to a regular or seasonally driven planktonic food source [24], [25]. These aggregations all occur close to coasts or reefs and are usually dominated by juvenile and sub-adult males [21], [26]–[29]. In most locations, research is based on the mark-recapture of photo-identified sharks. Each individual can be distinguished by their distinctive, persistent natural spot patterns [30], [31], and many have been shown to return in subsequent years to the same location [21], [28], [29], [32]. Satellite-linked pop-up archival and other satellite tags have been deployed to show that sharks are capable of significant long-distance movements, often through a series of political jurisdictions [16], [23], [33]–[39]. Given that coastal aggregation sites are characterised by size-and sexual-segregation, increased study of offshore sites is a key requirement to further conservation and management of the species [2]. Currently, little is known about the offshore occurrence of whale sharks, although Sequeira et al. [40] used data from oceanic purse-seine fleets to document their pelagic occurrence within the Indian Ocean.

There have been few previous records of whale sharks in the Arabian Gulf and adjacent seas. Data from inside the Arabian Gulf include encounters from Iraq [41] and Kuwait [42], while Brown [43] reported sightings from the UAE between 1987 and 1992, including five encounters inside the Arabian Gulf and one on the UAE east coast, with sharks of up to 10 m in length. In a 1981 demersal fisheries report relating to the Arabian Gulf and Gulf of Oman, Sivasubramaniam & Yesaki [44] listed the whale shark as an unmarketable species for the region. Beech [45] recorded a further encounter on the Arabian Gulf side of the UAE in 2002. Immediately outside the Arabian Gulf, White & Barwani [46], [47] reported several encounters from the Straits of Hormuz and Gulf of Oman, and Blegvad [48] had two encounters in Iranian waters in the Strait of Hormuz.

More recently however, with the large increase in populations of Gulf States and the associated growth in the SCUBA diving and boating community, there has been a marked increase in whale shark encounters, particularly by divers in the Musandam region of Oman. To access these data, the senior author established a regional public sightings initiative, “Sharkwatch Arabia”, in June 2010 to begin collating reports of whale shark sightings from the Arabian Gulf and adjacent waters. When anecdotal reports suggested that a previously unknown aggregation of up to 100 or more whale sharks was occurring during the boreal summer months in the Al Shaheen Oil field (S. Stig, pers. comm. 2010), approximately 90 km off the coast of Qatar in the Gulf, the opportunity was taken to investigate the occurrence of such a large aggregation in offshore waters. Here we provide details on the biological phenomena driving this aggregation, along with the numbers, temporal occurrence and population structure of sharks sighted at Al Shaheen.

## Materials and Methods

No specific permits were required for any part of this research. The project was carried out in conjunction with the Qatar Ministry of Environment. All research activities took place within Qatari waters and the sampling did not involve endangered or protected species.

### Study Area

**Figure 1:**
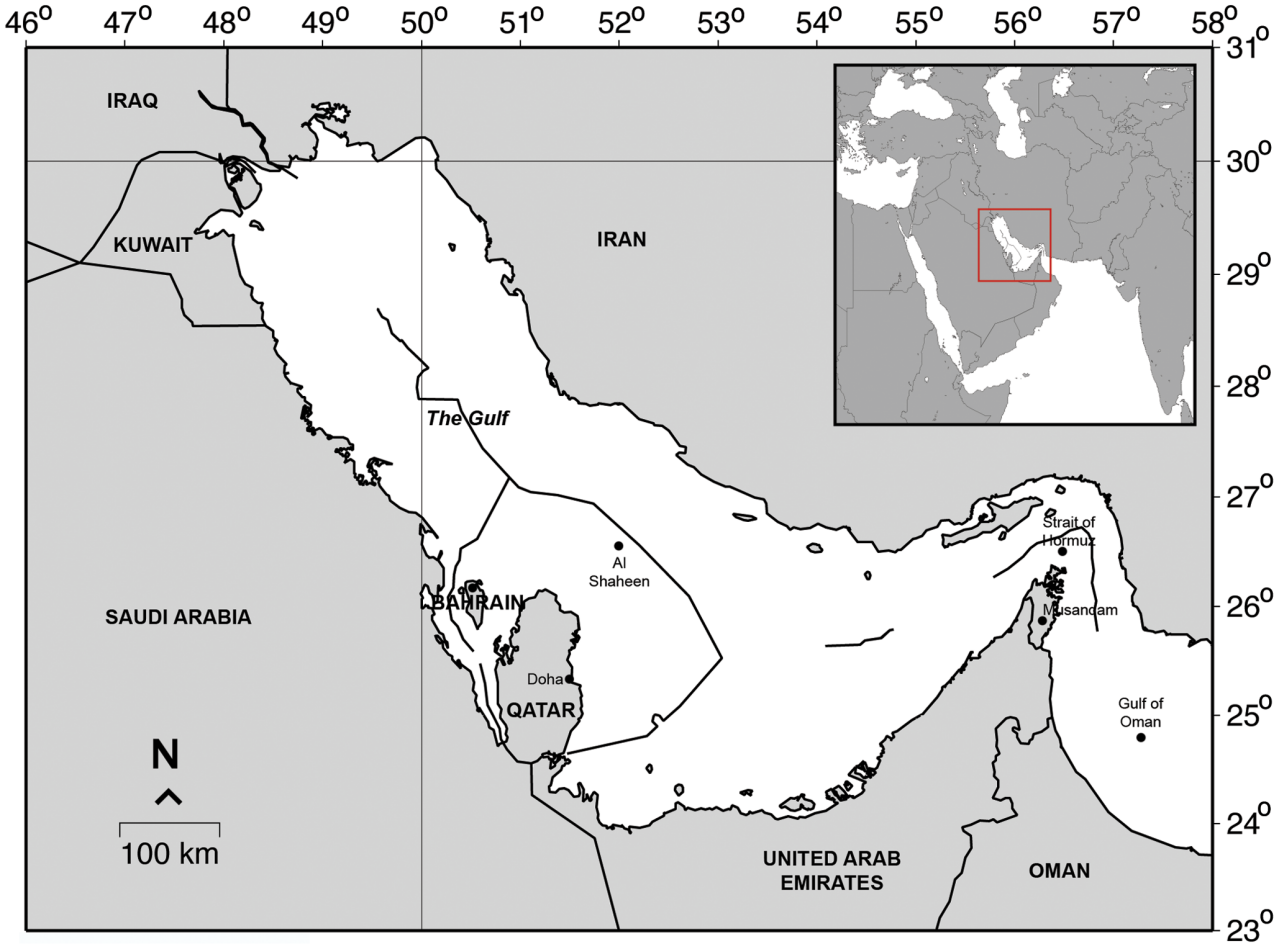
Map showing the respective locations of Qatar, the United Arab Emirates and the Al Shaheen oil and gas field within the Arabian Gulf, and (inset) of the Arabian Gulf itself in relation the Arabian Peninsula.

The Al Shaheen gas field lies approximately 145 km north of Doha, the Qatari capital, and 90 km offshore in the Arabian Gulf, which is a shallow, almost enclosed body of water with an average depth of 30 m (Figure 1). The Gulf is exposed to extreme environmental conditions, with sea surface temperatures regularly exceeding 35°C during summer and dropping below 15°C during the winter, while a lack of precipitation and high evaporation rates result in salinity in excess of 39 ppt [49]. Project surveys were undertaken in an area covering approximately 300 km^2^ of the gas field, bounded by eight fixed gas platforms.

### Platform Based Observations

Volunteer Maersk Oil staff, stationed on the platforms, provided reports of opportunistic observations of whale sharks. These sightings, often supported by video and photography, were logged throughout the May to December 2011 study period. The platforms are elevated, with 360° views to the water from most areas. All workers were briefed to report sightings and to record the time of the sighting along with the estimated number of individual sharks to their designated sightings collator. Only sharks reported during daylight hours were used in the final figures. One person stationed on each of the eight platforms was asked to collate sightings on a daily basis from their platform workers and log every time a shark or group of sharks was observed. Only the maximum number of sharks observed per day in one group was used in analysis so as to eliminate repeat observations. The website www.whaleshark.org was used to provide archived reports of whale shark occurrence in the region that occurred prior to the start of this study.

### Boat Based Observations

Eight boat-based surveys were conducted between 23^rd^ April 2011 and 8^th^ October, 2011. The surveys were carried out from a 10 m vessel powered by twin 250 cc engines, which took an average of two hours to reach the study area. Survey start times varied between 5 and 9 a.m. During each survey, a set route was followed from one to the next of the eight fixed gas platforms. Whale sharks were detected from sightings of the first dorsal and or caudal fin breaking the surface of the water. For logistical reasons no surveys could be conducted during August and September or during periods when wind speed exceeded 12 knots.

Upon sighting an individual or an aggregation of sharks, a GPS location and time were recorded and a team of between four and six researchers entered the water, using snorkelling gear and equipped with digital cameras. Researchers took photographs of the flank area on each shark behind the fifth gill slit and above the pectoral fin for the purposes of individual identification [30]. Photographs were also taken of any notable scars. Where possible, the size of each animal was estimated, usually by comparison with the boat or another snorkeler, and the sex of each animal was determined by the presence (males) or absence (females) of claspers. After completion of in-water observations, the numbers of whale sharks were estimated based on observations both in water and from the boat. Subsequently, images collected in the field were catalogued and then processed using I^3^S software [50].

### Plankton Sampling and Analysis

On each trip, one sample was taken at each fixed sample station using a 200 µm mesh net with 50 cm mouth diameter and attached flow meter, towed for three minutes at a speed of 1 to 2 knots. Wherever possible Conductivity-Temperature-Depth (CTD) casts were made and water temperature and salinity were recorded approximately 10 cm below the surface of the water (Table 1).

**Table 1:** Bio-volume, in-water surface temperature, salinity and numbers of organisms in plankton samples taken at fixed sampling stations and at sites where feeding whale sharks were encountered.

Date	Sampling Location	Bio-volume ml/l	Organisms per m^3^	# sharks	Surface Temp	Salinity
23-April-11	N/A	–	–	2	N/A	N/A
7-May-11	1	26.0	16421	0	24.5	39.4
7-May-11	Comparison	18.0	9976	–		
14-May-11	1	10.0	766	30	27.3	39.1
14-May-11	2	60.0	4725	–		
14-May-11	During Feeding	90.0	12447	–		
14-May-11	Comparison	12.0	1511	–		
29-May-11	1	40.0	8803	0	26.4	38.79
29-May-11	2	76.0	32214	–		
29-May-11	Comparison	8.0	7634	–		
7-Jun-11	1	28.0	18305	0	N/A	N/A
7-Jun-11	2	4.0	22160	–		
7-Jun-11	Comparison	8.0	22173	–		
25-Jun-11	1	50.0	13036	0	N/A	N/A
25-Jun-11	2	40.0	30019	–		
25-Jun-11	Comparison	60.0	23190	–		
9-Jul-11	1	24.0	40702	100	29.58	39.5
9-Jul-11	During feeding	95.0	19484	–		
9-Jul-11	Post feeding	50.0	13988	–		
8-Oct-11	1	64.0	56414	0	28.7	41.03
8-Oct-11	2	620.0	16904	–		
8-Oct-11	Comparison	7.5	24862	–		

Plankton were preserved in 4% buffered formalin. In addition, further surface plankton tows were conducted and replicate environmental data recorded whenever a shark or group of sharks was encountered. These “during feeding” plankton samples were taken at the most central location of the feeding group, and where possible the net towed in the same direction as the feeding sharks were swimming. In one instance, a “post-feeding” plankton sample was subsequently taken at the same GPS location after the sharks had moved away to determine whether plankton was still present in the area.

Three replicate sub-samples of 2 ml were transferred into petri dishes and the zooplanktonic organisms present identified and enumerated to species level under a compound microscope at 100× magnification using standard keys [51]–[54]. The mean of the three counts was used to estimate the numerical abundance of each zooplankton species. The volume of water filtered was calculated from the flow meter reading, and the approximate abundance and density of each species determined per cubic meter. The bio-volume of the zooplankton sample was determined using an adapted settlement method [55]; the volume of the original sample was increased to 1 litre and the plankton allowed to settle for 24 hours before the settlement value (ml/l) was recorded. Mann-Whitney *U* test was used to compare zooplankton bio-volume (ml/l) and organisms per m^3^ for samples taken within the study area and outside the study area at the comparison station.

### Genetic Identification of Fish Eggs

The “during feeding” plankton sampling methodology was modified following sampling on 14^th^ May, when high concentrations of fish eggs were found in the sample. This modified method involved immediate completion of a second plankton tow for one minute, with this second sample preserved in ethanol for COI (Cytochrome oxidase subunit 1) DNA barcoding to determine the fish species present.

Approximately 25 mg of each sample collected was sub-sampled and DNA was extracted (Qiagen kit: www.qiagen.com). The 650 bp COI barcode region was then amplified by Polymerase Chain Reaction (PCR) using a reaction mix (20 µl) containing Ampli Taq Gold 360 master Mix (Applied Biosystems; www.appliedbiosystems.com), 2 µL DNA template (concentration of 20 ng/µl) and 1 µl of each of two primers: BLCO11490 F (5′-GGT CAA CAA ATC ATA AAG ATA TTG G-3′) and BHCO2198R (5′-TAA ACT TCA GGG TGA CCA AAA AAT CA-3′). The cycling parameters were 35 cycles of 95°C/1 min, 40°C/1 min and 72°C/1 min 30 sec, with a final extension step at 72°C for 7 min, followed by cooling to 4°C (Folmer et al., 1994). PCR products were then visualized on 1.5% agarose gels.

Five µl of these PCR products were then purified using ExoSAp-iT Enzyme (USB; www.usbweb.com) according to the manufacturer procedure, following which they were labelled using a BigDye® Terminator v.3.1 Cycle Sequencing Kit (Applied Biosystem). The Big Dye PCR Products were then purified using a standard ethanol precipitation method (following Applied Biosystem standard protocol). The purified product from all samples was then sequenced bi-directionally using an ABI 3130 DNA sequencer (Applied Biosystem) and sequence trace files assembled using Sequencing Analysis v.5.1. The resulting sequences were then matched against individual specimens in the Barcode of Life (BOLD) database (www.bold.org).

## Results

### Archival Data

**Figure 2:**
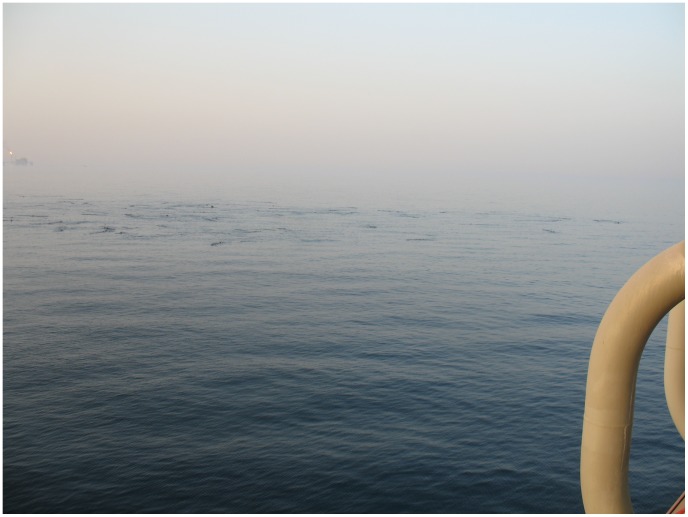
An image taken by Maersk Oil platform worker Soren Stig on 15^th^ August 2007, showing an aggregation of whale sharks feeding at the surface in the Al Shaheen Oil Field.

Seven previous encounters of whale sharks in Qatari waters had been reported to the global whale shark database (www.whaleshark.org) from individuals and oil platform workers in the region since 2004. Included in these seven reports are Soren Stig’s August 2007 report of a large whale shark aggregation in Al Shaheen (Figure 2) and a second report in May 2010 of an encounter with approximately 30 whale sharks off the coast of Qatar.

### Platform Based Observations

**Figure 3:**
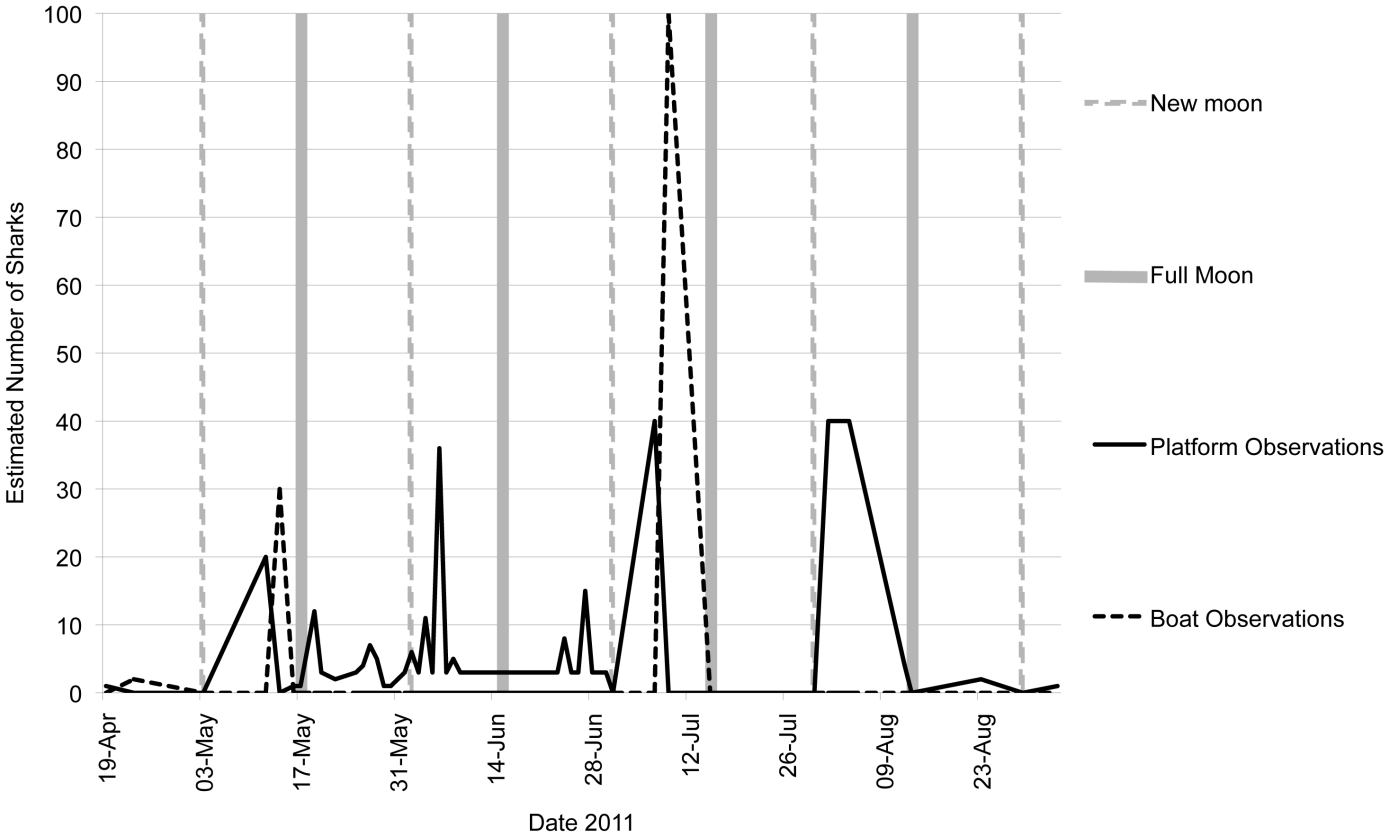
Estimated number of whale sharks seen during platform and boat observations and moon phase for May through September 2011.

Maersk platform workers frequently reported large numbers of whale sharks around the platforms between May and August (Figure 3), and three sharks in early September. No sharks were reported between October and December even though the number of platform workers remained the same. The maximum estimated number observed over a single month was 178, recorded during June. The maximum number of sharks observed by platform workers at any one time was estimated to be 40 animals.

### Boat Based Observations

Two individual whale sharks were encountered on 23^rd^ April 2011, both of which were swimming slowly at the surface with no feeding behaviour apparent. On subsequent dates when sharks were observed they were present in large groups, moving at speed with their mouths wide open, skimming the surface layer, actively ram feeding.

On 14^th^ May 2011, an estimated 30 sharks were encountered. Surface water temperatures were 27.3°C with a salinity of 39.1 ppt (Table 1). Due to the fast swimming speed of the feeding sharks it was difficult to obtain photographs of both left and right sides for photo-identification purposes. Up to 14 sharks were identified, with nine animals being photographed on the left and five on the right. Sex was determined for six of these individuals, two females and four males. Size estimates of the sharks varied between 6 m and 10 m. Significant scarring was noted on three (23%) of the identified individuals, with two bearing evidence of severe boat impact trauma distinguished by defined propeller marks on the body. No fresh wounds or scarring were observed, suggesting that the injuries were not recent. Ramírez-Macías et al [56] found 13–33% of whale sharks photographed near Holbox Island, Mexico, had significant scarring attributable to boat strikes. Although the percentage of scarring we observed fell within the range reported in the Holbox Island study, the number of whale sharks assessed for scarring from the 2011 season was low (14) and so further investigation into scarring is warranted. The sharks were observed feeding at a plankton bio-volume density of 90 ml/l (Table 1). The number of organisms (per m^3^) from fixed sampling within the study area varied throughout the sampling period between 766 and 56, 414 (mean 18844.48±13248.37). The “during feeding” sample taken on this occasion contained 12, 447 organisms (per m^3^), notably lower than the mean for the 2011 sampling season. This sample contained a high density of fish eggs.

On 9^th^ July 2011, an estimated 100 whale sharks were encountered under similar conditions. Surface water temperatures were 29.58°C with a salinity of 39.5 ppt (Table 1). One hundred and four identities were captured, 53 of left sides and 51 of right sides; only one shark was re-encountered from the 14^th^ May 2011 aggregation. Twenty-one male and four female sharks were sexed. Size was estimated for nine individuals and ranged between four and eight metres in length. High concentrations of fish eggs were present in the water column on both occasions. These sharks were observed feeding at a plankton bio-volume density of 95 ml/l. The number of organisms (per m^3^) from fixed sampling within the study area varied throughout the sampling period between 766 and 56, 414 (mean 18844.48±13248.37). The “during feeding” samples taken on this date contained 19, 484 organisms (per m^3^), not notably different from the mean; this sample also contained large quantities of fish eggs. The “post-feeding” sample contained 52.6% of the bio-volume (ml/l) and 71.8% of the number of organisms (per m^3^) than the “during feeding” sample taken four hours previously in the same location (Table 1). Samples of fish eggs from this day were subjected to COI barcoding and sequences compared to the Barcode of Life (BOLD) database (www.bold.org). Two separate sequences generated from independent egg samples matched mackerel tuna (*Euthynnus affinis*) with 100.0% similarity.

Fish eggs were the dominant plankton in the two samples taken “during feeding” by whale sharks (14^th^ May and 9^th^ July), accounting for 66.1% and 76.55% of the organisms present (Table 2). In no other samples did fish eggs account for more than 10.25%, except in the sample taken at station 2 on 8^th^ October. Samples of fish eggs from 8^th^ October were also subjected to COI barcoding and sequences compared to the Barcode of Life (BOLD) database (www.bold.org). Two separate sequences generated from independent egg samples matched Indian oil sardine (*Sardinella longiceps*) with 100.0% similarity.

**Table 2:** Results of taxonomic inspection of plankton samples showing for each sample the taxa of plankton which accounted for the largest, second largest and third largest portion of the plankton in terms of numbers of individuals, and for each taxa and for fish eggs, the percentage of the zooplankton by numbers for which they accounted.

Date	Sampling Location	Dominant Family	(%)	Second most dominant	(%)	Third most dominant	(%)	Fish eggs (%)
7-May-11	1	Radiolaria	68.33	Copepoda stages	17.33	Sagita spp.	2.66	0.33
7-May-11	Comparison	Copepoda stages	56.23	Appendicularia	13.14	Fish eggs	7.3	7.3
14-May-11	1	Radiolaria	38.6	Appendicularia	29.93	Sagita spp.	3.95	0
14-May-11	2	Appendicularia	21.8	Echinodermata larvae	21.8	Copepoda stages	26.11	0
14-May-11	Comparison	Protohabdonella spp.	23.69	Echinodermata larvae	23.69	Labidocera spp.	13.17	0
14-May-11	During Feeding	Fish eggs	66.1	Radiolaria	18.49	Copepoda stages	3.42	66.1
29-May-11	1	Radiolaria	85.96	Echinodermata larvae	12.08	Copepoda stages	0.83	0
29-May-11	2	Radiolaria	59	Appendicularia	16.66	Copepoda stages	4	3.6
29-May-11	Comparison	Radiolaria	42.4	Copepoda stages	28.48	Bivalve veligers	11.4	0
7-Jun-11	1	Radiolaria	78.8	Appendicularia	7.97	Cyclopoidae	4.41	1.7
7-Jun-11	2	Radiolaria	58.48	Appendicularia	17.49	Fish eggs	10.25	10.25
7-Jun-11	Comparison	Noctiluca	18.27	Copepoda stages	21.89	Appendicularia	16.87	1.61
9-Jul-11	1	Copepoda stages	33.86	Chaetognatha spp.	16.93	Calanoidae spp.	13.96	3.43
9-Jul-11	During feeding	Fish eggs	76.54	Copepoda stages	10.46	Calanoidae spp.	2.97	76.54
9-Jul-11	Post Feeding	Copepoda stages	30.82	Bivalve veligers	15.53	Appendicularia	10.82	3.06
8-Oct-11	1	Radiolaria	74.40	Appendicularia	5.03	Copepoda stages	2.95	2.79
8-Oct-11	2	Fish eggs	85.13	Radiolaria	10.77	Echinodermata Larvae	1.02	85.13
8-Oct-11	Comparison	Bivalve veligers	59.21	Copepoda stages	14.26	Calanoidae spp.	4.8	2.32

Bio-volume (ml/l) and number of organisms (per m^3^) from the comparison station taken outside the study area (n = 5) were found to be statistically different from samples taken at fixed sample station 1 (n = 7) and fixed sample station 2 (n = 5) located within the study area (Bio-volume: Mann-Whitney *U* test, p = 0.05; Organisms per m^3^: Mann-Whitney *U* test, p = 0.05).

Other species of marine megafauna were recorded during field surveys and by the platform workers. These included large pods of spinner dolphins (*Stenella longirostris*), Indo Pacific bottlenose dolphin (*Tursiops aduncus)*, hawksbill sea turtle (*Eretmochelys imbricata)*, green sea turtle (*Chelonia mydas)* and loggerhead sea turtle (*Caretta caretta)*, the Arabian sea snake (*Hydrophis lapemoides*), yellow bellied sea snake (*Pelamis platurus*), Cobia (*Rachycentrum canadum*), schools of scalloped hammerhead sharks (*Sphyrna lewini)* and other species of unidentified requiem sharks (*Carcharhinidae*).

## Discussion

A large number of whale sharks frequented the Al Shaheen Oil field from May through to September 2011. Concurrent plankton sampling suggested that this aggregation is related to mackerel tuna spawning events, although the results should not be considered conclusive due to the low sample size. The results of the statistical tests also suggest that the study area may have a higher overall productivity than open water outside of the study area, although the plankton sampling was not performed to specifically test this hypothesis. Observations from the boat surveys, platform workers combined with the plankton analysis results suggest that the platforms in the Al Shaheen area are also acting as offshore reefs and support an increased biodiversity compared to areas further from oil or gas platforms. Fish and crustacean spawning events have also been cited as a factor in the occurrence of whale sharks at a number of other sites, including Gladden Spit in Belize, Christmas Island in the Indian Ocean and Yucatan Peninsula in Mexico [15], [24], [25]. The Qatar aggregation seems to be similar in terms of density and behaviour of sharks to the “Afuera” aggregation (Figure S1) described to occur off the Yucatan Peninsula of Mexico. Whale sharks also aggregate to feed on tuna (little tunny *Euthynnus alletteratus)* spawn, in that location. Little tunny have also been linked to whale shark occurrence elsewhere [25], [40], [57].

Observations by both the platform workers and the authors suggest that the tuna are aggregating under or close to the platforms, and may be spawning in these locations which appear to be acting as fish aggregating devices (FADs). The tendency for tropical tuna to aggregate under floating structures is well-known, with half of the world’s tuna catch now obtained around FADs [58], [59]. The species to which the eggs belonged from 9^th^ July “during feeding” sample, as determined by the barcoding process, was the mackerel tuna. This species is a principal target of one FAD fishery in the Philippines [60]. It is thus possible that the large number of platforms in the field may in part be responsible for the large numbers of tuna spawning. Hoffmayer et al [61], similarly observed that the offshore platform areas in the Gulf of Mexico were acting as offshore reefs that were then frequented by whale sharks [62].

Mackerel tuna are migratory and widely distributed throughout the Indian Ocean [63]. Fisheries records confirm that this species occurs in Qatari waters [64], where it is believed to aggregate to spawn from May through to the end of August (M. Al-Jaidah, pers. obs.). Sivasubramaniam & Ibrahim [64] found that the largest specimens appeared in catches off Qatar between April and October, but they failed to record any females with ripe ovaries. Nevertheless the fact that the period when adults of this species are present in Qatari waters coincides with the apparent whale shark season supports the identity of the eggs of which the sharks are feeding. The last recorded whale shark by the platform workers in 2011 was in early September, and no whale sharks were observed feeding on high concentrations of Indian oil sardine *S. longiceps* eggs taken from the October 8^th^ sample. Sivasubramaniam. & Ibrahim [64] highlight that Al Shaheen is within a highly productive area for sardines, and it remains possible that the sharks also feed on the eggs of this or other fish species. It is not yet known why the sharks were not observed in the area beyond early September, even though there was still a potential source of food. Further plankton sampling taken at “during feeding” events is needed to build on the information collected here.

The specific temporal drivers of tuna spawning and resulting whale shark feeding aggregations, within the broader spring-summer season, remain unclear. Aggregations were encountered during boat surveys in May and July, but few surveys were possible during June and August. These were the peak months for whale shark sightings by platform-based observers with 178 and 166 sharks recorded, respectively. While at least some sharks were present throughout the May to September period, it appears likely that dense feeding aggregations occur as a specific response to tuna spawning. The lunar phase is known to have an effect on some fish spawning events and the subsequent occurrence of whale shark aggregations [34]. However, in this case, observations of whale sharks from the platforms occurred throughout the month, and sometimes over consecutive days, with no clear correlation with the phase of the moon. Mckinney et al [62] also noted numerous reports of whale shark observations from platform workers over consecutive days in the Gulf of Mexico.

Mean bio-volume (ml/l) was above average for the “during feeding” samples when large aggregations were encountered on 14^th^ May and 9^th^ July. Both of these samples were dominated by fish eggs, which, excluding the sample taken at station two on 8^th^ October, made up no more than 10.25% in any other sample. On 9^th^ July the “post feeding” plankton sample, taken at the same location four hours after intense feeding was observed but when the sharks were no longer feeding, showed a decline in the proportion of the sample made up of fish eggs from 76.54 to 3.06%. Soon after the “post feeding” sample was taken, the feeding sharks were observed 3 km south of where they had been observed four hours previously. The currents at the time were moving south so it is presumed the sharks were moving with drifting fish eggs in the water column.

Temperature and salinity were recorded within the Al Shaheen field of 27.3°C and 39.1 ppt on 14^th^ May, and 29.58°C and 39.5 ppt on 9^th^ July (Table 1). On both these dates, whale sharks were encountered within the area feeding at the surface. No temperature or salinity data was recorded in the field during the months of August or September when water temperatures in the Arabian Gulf are at their peak [49]. Platform workers frequently observed whale sharks throughout August (Figure 3) demonstrating that the sharks are able to tolerate the high temperature and salinity experienced in the Al Shaheen field during the summer months. Sequeira et al [40] used 17 years of archived whale shark sightings data from tuna purse-seine fisheries fleets around the Indian Ocean in an attempt to predict whale shark occurrence using variables including temperature. It was found that the whale sharks preferred a narrow band of temperature with 90% of sightings occurring between 26.5°C and 30°C and hypothesised that whale sharks may avoid higher temperatures as this may elevate metabolic rates and subsequently increase food requirements. The two aggregations that occurred in Al Shaheen on 14^th^ May and 9^th^ July fell within the range of temperature that Sequeira et al [40] found whale sharks to prefer. However, whale sharks were still occurring and feeding at the surface within the Al Shaheen area through August, the warmest month. A Maersk supply vessel recording water temperature within the field recorded that the temperature in the upper 10 m of the water column constantly exceeded 33°C throughout the entire month of August, reaching a maximum of 33.8°C on 12th August 2011 (S. Bach, pers. comm.). This shows that whale sharks are able to not only tolerate, but actively feed in temperatures of 33°C. As few marine habitats in the world experience the same extremes of environmental conditions as the Arabian Gulf, it may be here that the upper tolerance levels for whale sharks in terms of both temperature and salinity are to be found. The ability for whale sharks to tolerate these natural extremes of temperature and salinity, and how it may influence their ecology, is important to note in a changing global climate.

Strict standardisation of observer effort was not possible as a result of the fixed nature of platform-based surveys and the difficulty of access for boat-based surveys. While platform workers observed large numbers of whale sharks in months when they were undetected during the boat surveys, the ability of platform workers to estimate the number of sharks in the area is limited due to the static location of the platforms. Thus on 9^th^ July the boat survey estimated over 100 sharks present in one location, yet the platform workers reported only 40 sharks that week. Images submitted to the project’s website-based public sightings scheme “Sharkwatch Arabia” by an oil rig worker in Saudi Arabia document the occurrence on 6^th^ July of an aggregation of some 50 sharks approximately 130 km west of Al Shaheen. The whale sharks had been observed repeatedly at this site over the previous week, suggesting this may also be a regular aggregation site. Greater recruitment and training of platform workers to assist with the project, along with a more regular boat-based surveys and expansion to encompass aerial observations [25], [65], would help to provide a more accurate assessment of shark distribution and numbers. Given that the peak timing of whale shark observations in the Straits of Hormuz and Gulf of Oman is in April and November [47], [48], slightly before/after the Qatari aggregation, it is also plausible that sharks may leave the Gulf and travel more broadly within the Arabian Sea.

The large numbers of whale sharks observed at Al Shaheen and comparisons with numbers from other known aggregations [14]–[23] indicate that this area is a major aggregation site for the species. Mckinney et al [62] highlight that the association between whale sharks and offshore platforms warrants further investigation. Here we suggest that the whale sharks aggregating in the Al Shaheen area are indirectly associated with the platforms through the presence of spawning tuna (Figure S2). The small number of boat-based surveys possible over the course of this study, coupled with the fast swimming speed and erratic movements of feeding sharks, made efforts to photo-identify individual sharks difficult. Nevertheless, 63 left side identities and 56 right side identities were collected overall, with only one individual encountered on more than one occasion (14^th^ May and 9^th^ July). Vessel access is restricted in the area and, for operational and security reasons, there is a 500 m restricted zone around all platforms (S. Bach, pers. comm.). Thus, as well as favouring spawning of tuna, the oil field may be effectively serving as a sanctuary for the whale sharks, as they are less prone to disturbance. It is hoped that further consultation with the Qatar Ministry of Environment and Maersk Oil will lead to proposals for sustaining the marine life of this unintended protected area.

A recent review of whale shark biology and ecology [2] highlighted the need to determine whether aggregations of individuals occur at offshore sites, as well as in the coastal locations where in recent years they have become well studied [14], [15], [23]–[25], [27]. In particular the future identification of hitherto unknown offshore aggregation locations might help provide a rationale for the species’ trans-oceanic foraging. The observations presented here clearly demonstrate that aggregations of these animals can occur at a site as far as 90 km offshore (but still shallow: 60 m deep); however it remains to be considered whether the aggregation is essentially similar in character from those described for more coastal locations where the sharks also aggregate in response to a seasonally available high-value food source [14], [15], [23]–[25], [27].

## Supporting Information

Figure S1 **An aerial image of a whale shark aggregation in the Al Shaheen area showing typical density of feeding sharks and variation in size (image taken by Mohammed Y. Jaidah).**
(TIF)

Figure S2 **A split level image showing a whale shark in close proximity to an offshore platform in the Al Shaheen area (image captured by Warren Baverstock).**
(TIF)
