# Supplementary figures and images for: Whale Sharks, Rhincodon typus, Aggregate around Offshore Platforms in Qatari Waters of the Arabian Gulf to Feed on Fish Spawn
Source: PLoS One. 2013 Mar 13;8(3):e58255. doi: 10.1371/journal.pone.0058255 (PMC3596407; doi:10.1371/journal.pone.0058255)

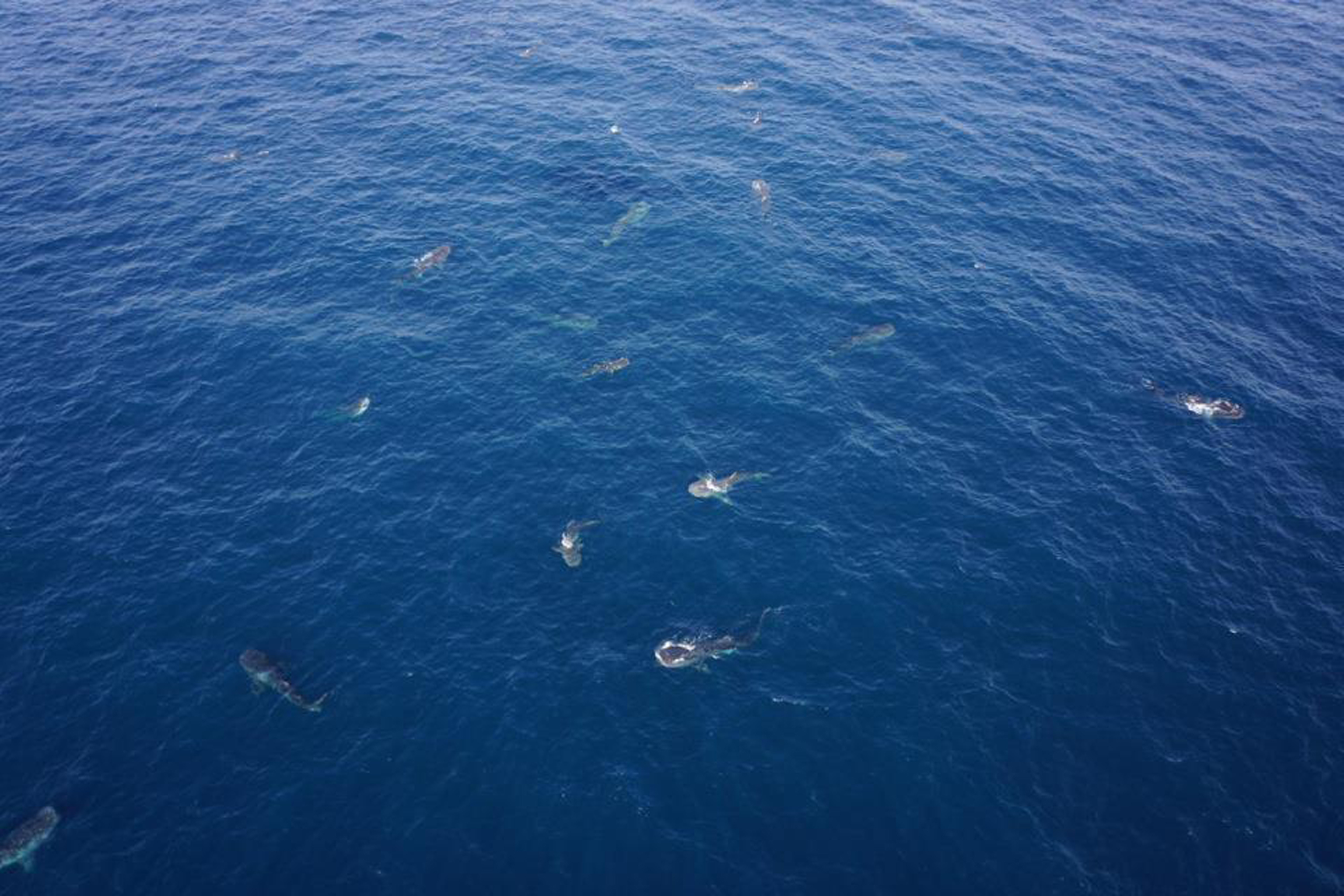

Supplement: Figure S1 — An aerial image of a whale shark aggregation in the Al Shaheen area showing typical density of feeding sharks and variation in size (image taken by Mohammed Y. Jaidah). (TIF) [file pone.0058255.s001.tif]

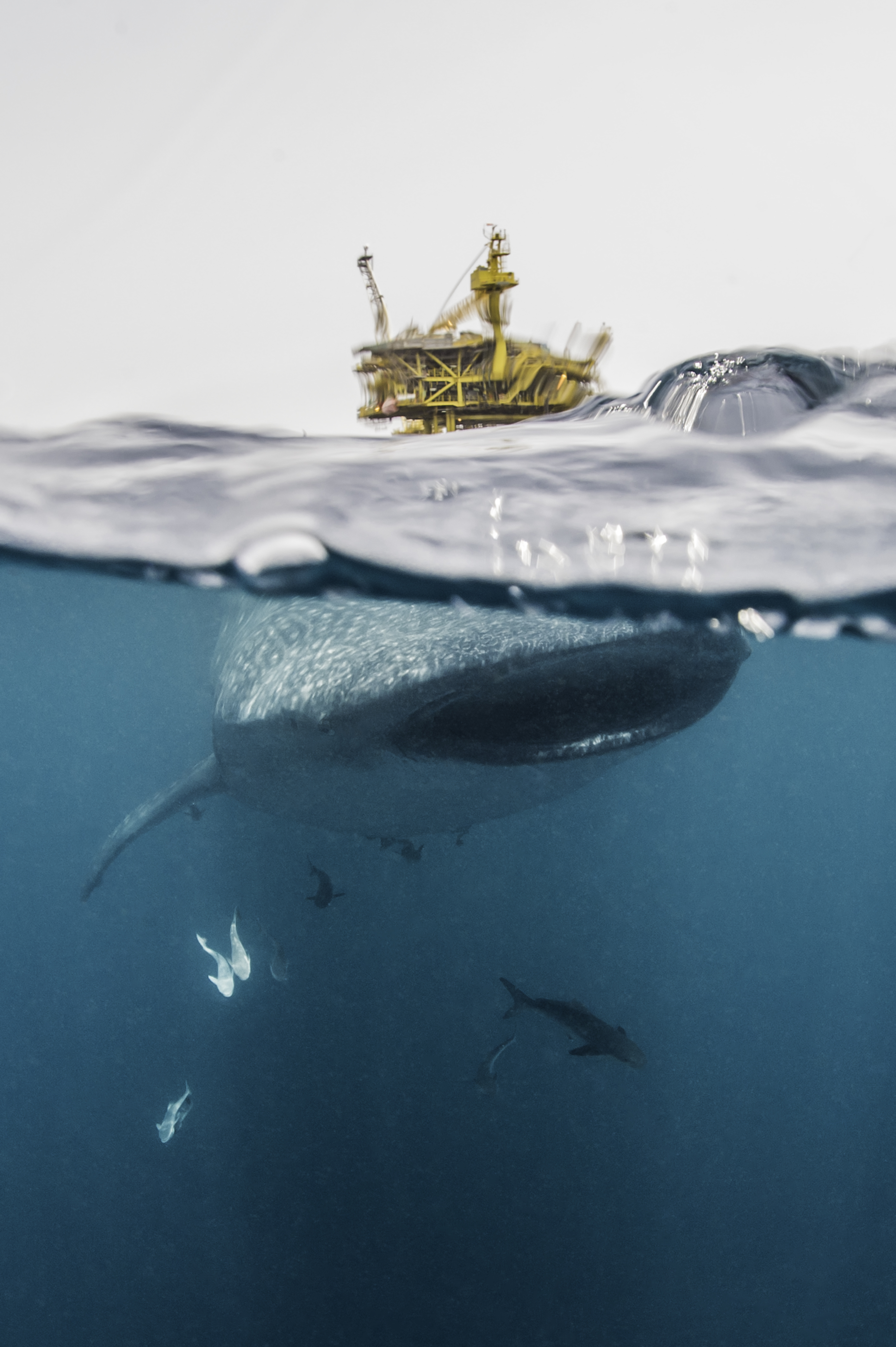

Supplement: Figure S2 — A split level image showing a whale shark in close proximity to an offshore platform in the Al Shaheen area (image captured by Warren Baverstock). (TIF) [file pone.0058255.s002.tif]
